# Association of Deceased Donor Acute Kidney Injury With Recipient Graft Survival

**DOI:** 10.1001/jamanetworkopen.2019.18634

**Published:** 2020-01-08

**Authors:** Caroline Liu, Isaac E. Hall, Sherry Mansour, Heather R. Thiessen Philbrook, Yaqi Jia, Chirag R. Parikh

**Affiliations:** 1 Division of Nephrology, Department of Medicine, The Johns Hopkins University School of Medicine, Baltimore, Maryland; 2 Division of Nephrology and Hypertension, Department of Internal Medicine, University of Utah School of Medicine, Salt Lake City; 3 Program of Applied Translational Research, Section of Nephrology, Yale University School of Medicine, New Haven, Connecticut

## Abstract

**Importance:**

The shortage of deceased donor kidneys for transplants is an ongoing concern. Prior studies support transplanting kidneys from deceased donors with acute kidney injury (AKI), but those investigations have been subject to selection bias and small sample sizes. Current allocation practices of AKI kidneys in the United States are not well characterized.

**Objectives:**

To evaluate the association of deceased donor AKI with recipient graft survival and to characterize recovery and discard practices for AKI kidneys by organ procurement organizations.

**Design, Setting, and Participants:**

Registry-based, propensity score**–**matched cohort study from January 1, 2010, to December 31, 2013, in the United States. The dates of analysis were March 1 to November 1, 2019. From 2010 to 2013, a total of 6832 deceased donors with AKI and 15 310 deceased donors without AKI had at least 1 kidney transplanted. This study used a 1:1, propensity score**–**matched analysis to match deceased donors with AKI to deceased donors without AKI and investigated outcomes in their corresponding kidney recipients.

**Exposure:**

Deceased donor AKI, defined as at least 50% or 0.3-mg/dL increase in terminal serum creatinine level from admission.

**Main Outcomes and Measures:**

Recipients were assessed for the time to death-censored graft failure and the following secondary outcomes: delayed graft function, primary nonfunction, and the time to all-cause graft failure.

**Results:**

Ninety-eight percent (6722 of 6832) of deceased donors with AKI were matched to deceased donors without AKI. The mean (SD) age of the 13 444 deceased donors was 40.4 (14.4) years, and 63% (8529 of 13 444) were male. A total of 25 323 recipients were analyzed (15 485 [61%] were male), and their mean (SD) age was 52.0 (14.7) years. Recipients were followed up for a median of 5 (interquartile range, 4-6) years. Deceased donor AKI status had no association with death-censored graft failure (hazard ratio, 1.01; 95% CI, 0.95-1.08) or all-cause graft failure (hazard ratio, 0.97; 95% CI, 0.93-1.02). The results were consistent after examining by AKI stage and adjusting for recipient and transplant characteristics. More recipients of AKI kidneys developed delayed graft function (29% vs 22%, *P* < .001). Few recipients (120 of 25 323 [0.5%]) developed primary nonfunction regardless of deceased donor AKI status. Recovery and transplantation of AKI kidneys varied by organ procurement organization; most (39 of 58) had high recovery and high discard of AKI kidneys.

**Conclusions and Relevance:**

Deceased donor AKI kidneys transplanted in the study period had recipient graft survival comparable to that of non-AKI kidneys. This study’s findings suggest that the transplant community should evaluate whether currently discarded AKI kidneys from donors without substantial comorbidities can be used more effectively.

## Key Points

**Question:**

Is deceased donor acute kidney injury (AKI) associated with recipient graft survival after matching on deceased donor AKI propensity?

**Findings:**

In this registry-based, propensity score**–**matched cohort study of deceased donors with and without AKI, deceased donor AKI had no independent association with short-term and long-term recipient graft survival. Recovery and transplantation of AKI kidneys varied by organ procurement organization; most (39 of 58) had high recovery and high discard of AKI kidneys.

**Meaning:**

This study’s findings suggest that the transplant community should continue to use deceased donor AKI kidneys and consider research to investigate whether currently discarded AKI kidneys can be used more effectively.

## Teaser

This cohort study evaluates the association of deceased donor acute kidney injury with recipient graft survival and characterizes recovery and discard practices for kidneys with acute kidney injury by organ procurement organizations.

## Introduction

The shortage of deceased donor kidneys has been an ongoing concern in the field of kidney transplantation. As a result, the use of kidneys with certain risk factors (eg, kidneys from older donors or donors with HIV or hepatitis C) has increased in an effort to meet the demands of the waiting list.^1^ As of October 2019, approximately 95 000 patients with end-stage renal disease (ESRD) who cleared medical evaluations remained on the waiting list.^2^ Approximately 9000 patients are removed from the waiting list every year because of death or deteriorating health, indicating that the organ shortage is far from resolved despite increased use of kidneys from higher-risk deceased donors.^3^

In 2019, the Advancing American Kidney Health^4^ initiative was launched with the following 3 primary aims: to reduce the number of patients developing kidney failure, to decrease the number of US individuals receiving dialysis in dialysis centers, and to increase the number of kidneys available for transplant. In particular, the initiative aims to “double the number of kidneys available for transplant by 2030,”^4^^(p3)^ which will require substantial changes to the organ procurement system.

Kidneys from deceased donors with acute kidney injury (AKI) may help reduce the kidney shortage. In some clinical settings (eg, sepsis and cardiac surgery), AKI and chronic kidney disease (CKD) have been hypothesized to be interconnected syndromes.^5^ However, AKI in the setting of deceased donor transplant may not be comparable. A variety of clinical actions can lead to serum creatinine (Scr) level fluctuations that meet criteria for AKI in deceased donors who typically do not have CKD, severe cardiovascular disease, or sepsis. In particular, vasopressin fluctuations during brain death can contribute to fluid losses and a number of hemodynamic changes,^6^ which do not necessarily lead to the intrinsic tissue injury commonly associated with CKD development.^7,8,9^ Kidneys procured from deceased donors may also have greater repair potential because the donors are typically younger and have fewer comorbidities than patients who experience AKI in settings in which the AKI leading to the CKD paradigm has been developed.^10,11,12^ Literature on repair biomarkers in the deceased donor setting suggests that donor injury can initiate repair processes and ischemic preconditioning, which may be beneficial at the time of transplant in the recipient.^13^ Taken together, kidneys from deceased donors with AKI could expand the pool of available kidneys for transplants.

Current kidney transplant practices are limited in their ability to characterize deceased donor AKI. In 2014, the United Network for Organ Sharing (UNOS) implemented a new Kidney Allocation System (KAS). Through the KAS, deceased donor organ quality is quantified through the Kidney Donor Profile Index (KDPI), which provides greater granularity over prior binary classifications of expanded vs standard criteria donors.^14,15^ A limitation of the KDPI is that it is restricted to the terminal Scr level at the time of organ procurement. A single creatinine level cannot differentiate deceased donor AKI from preexisting CKD. Furthermore, the terminal Scr level obscures much of the heterogeneity of underlying etiologies that contributed to kidney injury.^16^

Evidence from single-center and multicenter cohorts largely supports the use of selected kidneys from deceased donors with AKI.^17,18,19,20,21,22,23,24,25,26^ Despite the overall evidence supporting transplants of AKI kidneys, current allocation practices of AKI kidneys by organ procurement organizations (OPOs) have not been well characterized. Deceased donor AKI has been shown to be strongly associated with kidney discard,^18^ although that study was limited to only 5 OPOs and does not represent national practices. Furthermore, relevant cohort studies^27,28,29,30^ are limited by selection bias and confounders. Because a true clinical trial evaluating the role of deceased donor AKI in recipient outcomes is infeasible, we mimicked a clinical trial using registry data from DonorNet and the Organ Procurement and Transplantation Network (OPTN) to match deceased donors with and without AKI and to evaluate the association of deceased donor AKI with recipient graft survival. We then characterized current recovery and discard practices of AKI kidneys across the United States.

## Methods

### Propensity Score**–**Matched Analysis Cohort

This US registry-based, propensity score**–**matched cohort study was performed from January 1, 2010, to December 31, 2013. The dates of analysis were March 1 to November 1, 2019. From 2010 to 2013, a total of 6832 deceased donors with AKI and 15 310 deceased donors without AKI had at least 1 kidney transplanted. We used DonorNet data from the OPTN system, which registers deceased donors and communicates organ offers. The OPTN system includes data submitted by OPTN members on all donors, wait-listed candidates, and transplant recipients in the United States. The Health Resources and Services Administration (HRSA) of the US Department of Health and Human Services provides oversight to the activities of the OPTN contractor. The OPTN data from 2010 to 2013 have previously been validated with medical record–abstracted data for delayed graft function (DGF) and recipient graft survival from 5 OPOs.^31^ Validation of serial Scr levels from DonorNet with medical record–abstracted Scr levels from the same 5 OPOs is summarized in eTable 1 in the Supplement. The Kidney Donor Risk Index and KDPI were retrospectively calculated relative to all deceased donors in 2010 in the OPTN database.^32,33^ The study was approved by the HRSA and the institutional review boards at participating institutions under a waiver of consent because deidentified data were used. The clinical and research activities reported herein are consistent with the Principles of the Declaration of Istanbul as outlined in the Declaration of Istanbul on Organ Trafficking and Transplant Tourism.

**Figure 1.**
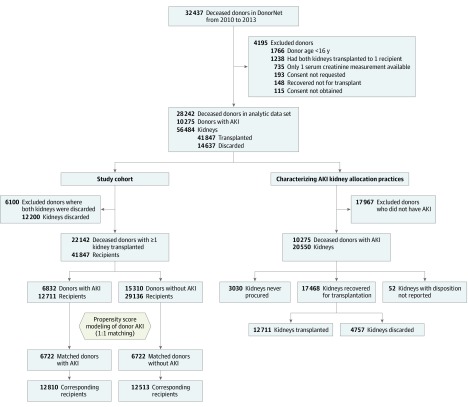
Flowchart of Study Cohorts Among study cohort members listed at the bottom of the figure, the median follow-up time was 4.8 (interquartile range, 3.5-6.0) years for 12 810 recipients from deceased donors with acute kidney injury (AKI) and 4.8 (interquartile range, 3.6-6.0) years for 12 513 recipients from deceased donors without AKI.

Of the available 32 437 deceased donors in DonorNet from 2010 to 2013, we excluded donors with only 1 Scr level available (n = 735), donors who had both kidneys transplanted to 1 recipient (n = 1238), donors younger than 16 years (n = 1766), donors whose informed consent was not available (n = 308), and donors whose kidneys were recovered for reasons other than transplant (n = 148), for a total analytic data set of 28 242 deceased donors. We then further excluded donors who had both kidneys discarded (n = 6100), for a final analytic sample of 22 142 donors, 6832 of whom had AKI (Figure 1). The encrypted UNOS donor identification numbers provided by DonorNet were then linked to the UNOS Standard Transplant Analysis and Research (STAR) files to link donors with their corresponding kidney recipients and UNOS-defined recipient outcomes. The analyses were based on DonorNet data submitted as of January 2017 and STAR data as of May 2018. We adhered to the ethics principles of the Declaration of Helsinki.^34^ This study followed the Strengthening the Reporting of Observational Studies in Epidemiology (STROBE) reporting guideline.

### Defining Deceased Donor AKI and Outcomes

DonorNet provides multiple serial measurements of the Scr level within a given date during hospitalization for each donor. We used the lowest Scr level on the hospital admission date and the highest Scr level on the terminal date (date of nephrectomy). Deceased donor AKI was defined as at least a 50% increase in the terminal Scr level from admission or an absolute increase in the terminal Scr level of at least 0.3 mg/dL (to convert Scr level to micromoles per liter, multiply by 88.4), irrespective of urine output (because it was not available in the data set). Stages of AKI were defined by KDIGO^35^ (Kidney Disease: Improving Global Outcomes) Scr level criteria as follows: stage 1 (≥0.3 mg/dL or 50% increase from admission to the terminal Scr level), stage 2 (100% increase from admission to the terminal Scr level), and stage 3 (>4.0 mg/dL or 200% increase from admission to the terminal Scr level), irrespective of urine output or initiation of dialysis because these data were not available.

The primary outcome of interest was the time to death-censored graft failure (dcGF) from the transplant date. Secondary outcomes of interest were DGF, primary nonfunction (PNF), 6-month renal function, and the time to all-cause graft failure (GF). Delayed graft function was defined as any dialysis in the first week after transplant. Primary nonfunction was defined per UNOS as kidney graft removal or as any dialysis or a creatinine clearance or estimated glomerular filtration rate (eGFR) of 20 mL/min/1.72 m^2^ or less, within the first 90 days after the first week. Six-month eGFR used the Scr level reported on the UNOS 6-month follow-up form^31^ and was calculated with the Chronic Kidney Disease Epidemiology Collaboration (CKD-EPI) equation.^36^ All-cause GF was defined as all-cause mortality, return to dialysis, or retransplantation.

### Statistical Analysis

We completed a propensity score–matched analysis in which the propensity score was composed of deceased donor characteristics of age, sex, race, body mass index, hypertension, diabetes, donation after cardiovascular determination of death, admission Scr level, and hepatitis C seropositivity. Deceased donors with AKI were propensity score matched 1:1 to deceased donors without AKI based on the logit of the estimated propensity score using a greedy matching algorithm. The matching used a caliper of 0.2 of the SD of the logit of the estimated propensity score.^37^ We examined standardized differences and created plots to assess the balance of continuous baseline covariates to investigate whether the propensity score was adequate.

Continuous variables were reported as the median (interquartile range [IQR]) or the mean (SD). Differences in demographic characteristics, clinical characteristics, and recipient outcomes by deceased donor AKI were compared using standardized differences.^38,39^

We used Cox proportional hazards regression models to assess the associations of deceased donor AKI with dcGF and all-cause GF. We adjusted Cox proportional hazards regression models for cold ischemia time and the following recipient characteristics: age, sex, black race, diabetes as the cause of recipient ESRD, preemptive transplant (a transplant before the initiation of dialysis), previous kidney transplant, HLA mismatch level, panel reactive antibody (percentage), and body mass index. The proportional hazards assumption was evaluated by the Kolmogorov-type supremum test.^40^ Because a donor may have 1 or 2 recipients, we estimated 95% CIs using robust sandwich estimates to account for the paired nature of the data.^41^

With a sample size of approximately 20 000 recipients of whom 30% received kidneys from deceased donors with AKI, we had more than 90% power to detect a hazard ratio (HR) of 1.15 with an overall event rate of 14%.^42,43^ As a confirmatory analysis, we conducted an unmatched analysis of the entire cohort using inverse probability treatment weighting of the propensity score.^44^

All inference testing was 2-sided with a significance level of *P* < .05. Analyses were conducted in SAS, version 9.4 (SAS Institute Inc) and Stata, version 14 (StataCorp LLC).

### Characterizing AKI Kidney Allocation Practices

To characterize allocation practices for AKI kidneys at the OPO level, we examined the proportion of AKI kidneys recovered and the proportion of AKI kidneys transplanted. To examine AKI kidney recovery patterns, we restricted the analytic data set of 28 242 deceased donors to those with AKI, for a final analytic sample of 10 275 deceased donors with AKI, yielding 20 550 kidneys available for recovery (Figure 1). We calculated the proportion of AKI kidneys recovered among the kidneys available by each OPO. We then calculated the discard proportion for each OPO, defined as the proportion of kidneys that were not transplanted after recovery. We mapped recovery and discard proportions by OPO and their corresponding donation service areas. The lowest tertiles of recovery and discard became cutoffs that were then combined to represent the following 4 categories of recovery and discard: low recovery and low discard, low recovery and high discard, high recovery and low discard, and high recovery and high discard.

To examine AKI kidney transplant patterns, we looked at the analytic data set of 41 487 kidneys transplanted. We calculated the proportion of AKI kidneys transplanted at both the OPO level and the transplant center level and then mapped the results.

Map files containing donation service area shapefiles were accessed from HRSA map services (https://gis.hrsa.gov/arcgis/rest/).^45^ All maps were created using ArcGIS Pro (version 2.20; Esri).

## Results

We matched 98% (6722 of 6832) of deceased donors with AKI to deceased donors without AKI (Figure 1). The mean (SD) age of the 13 444 deceased donors was 40.4 (14.4) years, and 8529 (63%) were male. A total of 25 323 recipients were analyzed (15 485 were male [61%]), and their mean (SD) age was 52.0 (14.7) years. Recipients were followed up for a median of 5 (IQR, 4-6) years. Insubstantial differences in deceased donor characteristics were consistent with the propensity score–matched analysis (Table 1). Deceased donor characteristics of the propensity score–matched cohort and the entire study cohort are listed in eTable 2 in the Supplement, with no meaningful differences. Matching criteria characteristics among unmatched deceased donors are listed in eTable 3 in the Supplement. The cumulative distribution functions and quantile-quantile plots (eFigure 1 in the Supplement) further confirm the balance of baseline continuous covariates (age, body mass index, and admission Scr level) between deceased donors with and without AKI.

**Table 1.** Demographic and Clinical Characteristics of Propensity Score–Matched Deceased Donors and Corresponding Recipients

Variable	Deceased Donors, No. (%)		Standardized Difference, %
	Without AKI (n = 6722)	With AKI (n = 6722)	
**Deceased Donor Matching Characteristics**			
Age, median (IQR), y	42 (27-52)	42 (28-52)	0.5
Male sex	4265 (63)	4264 (63)	0
Black race	1324 (20)	1227 (18)	3.7
BMI, median (IQR)	27.27 (23.65-31.85)	27.18 (23.67-31.64)	2.7
Hypertension	2204 (33)	2191 (33)	0.4
Diabetes	572 (9)	579 (9)	−0.4
Donation after cardiovascular determination of death	569 (8)	487 (7)	4.5
Expanded criteria donor^a^	1216 (18)	1316 (20)	−3.8
Hepatitis C seropositivity	155 (2)	155 (2)	0
Admission serum creatinine level, median (IQR), mg/dL	0.9 (0.7-1.2)	0.9 (0.7-1.2)	1.1
**Additional Deceased Donor Characteristics**			
CDC high-risk status	716 (11)	786 (12)	−3.3
Acute Kidney Injury Network stage^b^			
Without AKI	6722 (100)	NA	NA
Stage 1	NA	4621 (69)	NA
Stage 2	NA	1409 (21)	
Stage 3	NA	692 (10)	
Duration of AKI, median (IQR), d	NA	2 (1-3)	NA
Terminal serum creatinine level, median (IQR), mg/dL	0.8 (0.7-1.0)	1.5 (1.1-2.0)	−96.4
No. of kidneys transplanted			
1	634 (9)	930 (14)	−13.8
2	6088 (91)	5792 (86)	13.8
Kidney Donor Risk Index, median (IQR)	1.14 (0.90-1.46)	1.25 (1.01-1.57)	−27.3
Kidney Donor Profile Index, %			
Median (IQR)	41 (19-66)	51 (30-73)	−30.6
0-19	1744 (26)	870 (13)	33.3
20-49	2201 (33)	2355 (35)	−4.8
50-84	2194 (33)	2623 (39)	−13.3
85-100	583 (9)	874 (13)	−14.0
Cause of death			
Anoxia	1478 (22)	1708 (25)	−8.1
Cerebrovascular or stroke	2353 (35)	2544 (38)	−5.9
Head trauma	2692 (40)	2315 (34)	11.6
Other	199 (3)	155 (2)	4.1
Time from donor admission to organ recovery, median (IQR), d	3 (2-5)	3 (2-5)	−2.5
**Recipient Characteristics**			
No.	12 810	12 513	NA
Age, y			
<18	525 (4)	291 (2)	10.1
18-29	627 (5)	552 (4)	2.3
30-39	1375 (11)	1309 (11)	0.9
40-49	2520 (20)	2416 (19)	0.9
50-59	3488 (27)	3386 (27)	0.4
≥60	4275 (33)	4559 (36)	−6.4
Male sex	7821 (61)	7664 (61)	−0.4
Black race	4071 (32)	4109 (33)	−0.0
Wait time			
<6 mo	2521 (20)	2271 (18)	3.9
6 mo to <2 y	3778 (29)	3549 (28)	2.5
≥2 y	6511 (51)	6693 (54)	−5.3
Cause of recipient ESRD			
Diabetes	4085 (32)	3938 (32)	0.9
Hypertension	2884 (23)	2977 (24)	−3.0
Glomerulonephritis	2085 (16)	1994 (16)	0.9
Graft failure	945 (7)	817 (7)	3.3
Other or unknown	2811 (22)	2787 (22)	−0.8
Preemptive transplant	1595 (12)	1431 (11)	3.1
Previous kidney transplant	1556 (12)	1362 (11)	4.0
Pretransplant blood transfusion	2361 (18)	2329 (19)	−0.5
HLA mismatch level^c^			
0	900 (7)	838 (7)	1.3
1	128 (1)	114 (1)	0.9
2	503 (4)	483 (4)	0.3
3	1592 (12)	1594 (13)	−0.9
4	3362 (26)	3281 (26)	0
5	4133 (32)	4094 (33)	−1.0
6	2103 (16)	2020 (16)	0.7
Panel reactive antibody, %			
0-9	8640 (67)	8651 (69)	−3.6
10-39	1161 (9)	1142 (9)	−0.2
40-84	1573 (12)	1439 (12)	2.4
≥85	1436 (11)	1281 (10)	3.1
ESRD duration, median (IQR), mo	42 (22-66)	44 (23-68)	−3.7
Kidney biopsied	5346 (42)	7299 (58)	−33.7
Kidney pumped	4044 (32)	4708 (38)	−12.8
Cold ischemia time, median (IQR), h	14.33 (9.88-20.02)	16.00 (11.00-22.40)	−20.9

Abbreviations: AKI, acute kidney injury; BMI, body mass index (calculated as weight in kilograms divided by height in meters squared); CDC, Centers for Disease Control and Prevention; ESRD, end-stage renal disease; IQR, interquartile range; NA, not applicable.

SI conversion factor: To convert serum creatinine level to micromoles per liter, multiply by 88.4.

^a^ Defined as donor 60 years or older or 50 to 59 years with 2 or more of the following risk factors: hypertension, terminal serum creatinine level exceeding 1.5 mg/dL, and cerebrovascular or stroke cause of death.

^b^ Urine output criteria and initiation of donor dialysis were unavailable and not used in the definition for AKI and stages of AKI.

^c^ Data on HLA mismatch level are missing in 178 recipients (0.7%).

Most of the 6772 deceased donors with AKI had stage 1 (4621 [69%]), followed by stage 2 (1409 [21%]) and stage 3 (692 [10%]). Deceased donors without AKI were more likely to have both kidneys transplanted than deceased donors with AKI (6088 of 6722 [91%] vs 5792 of 6722 [86%]; standardized difference, 14%). Additional deceased donor and recipient characteristics stratified by stages of AKI are listed in eTable 4 in the Supplement.

Follow-up time was similar among recipients regardless of deceased donor AKI status as follows: 4.77 (IQR, 3.56-5.92) years for no AKI, 4.82 (IQR, 3.56-5.96) years for stage 1, 4.76 (IQR, 3.48-5.95) years for stage 2, and 4.77 (IQR, 3.49, 5.89) years for stage 3. Duration of follow-up, recipient sex, black race, and HLA mismatch level were comparable by deceased donor AKI status (Table 1). Recipients of kidneys from deceased donors with AKI were marginally older, experienced increased wait times, were more likely to have undergone prior transplants, and had longer ESRD duration (Table 1). Kidneys from deceased donors with AKI were more often biopsied and/or pumped before transplant and had longer cold ischemia times. Kidneys from deceased donors with stage 3 AKI were more likely to be biopsied and pumped than kidneys from deceased donors without AKI or deceased donors with lesser stages of AKI (Table 1).

### Recipient Graft Survival

Among recipients of kidneys from deceased donors with AKI, 29% (3643 of 12 513) developed DGF compared with 22% (2779 of 12 810) of recipients of kidneys from deceased donors without AKI (relative risk, 1.34; 95% CI, 1.28-1.41; *P* < .001) (Table 2). Delayed graft function was more common with increasing stages of deceased donor AKI as follows: 25% (2157 of 8627) for stage 1, 32% (838 of 2613) for stage 2, and 51% (648 of 1273) for stage 3. Few recipients developed PNF (120 of 25 323 [0.5%]) regardless of deceased donor AKI status. There was a statistically significant difference (*P* = .03) but no clear pattern in the proportion of recipients who developed PNF by stages of AKI, with rates of 63 (0.5%), 32 (0.4%), 12 (0.5%), and 13 (1.0%) for no AKI, stage 1, stage 2, and stage 3, respectively. Six-month renal function was statistically significantly different (*P* < .001) but not meaningfully different across stages of AKI, with mean (SD) 6-month eGFRs of 61 (22), 58 (22), 57 (22), and 62 (22) mL/min/1.73 m^2^ for no AKI, stage 1, stage 2, and stage 3, respectively.

**Table 2.** Delayed Graft Function and Primary Nonfunction by Deceased Donor AKI Status

Variable	Deceased Donors		Stage of AKI			*P* Value^a^
	Without AKI (n = 12 810)	With AKI (n = 12 513)	1 (n = 8627)	2 (n = 2613)	3 (n = 1273)	
Delayed graft function, No. (%)	2779 (22)	3643 (29)	2157 (25)	838 (32)	648 (51)	<.001
Primary nonfunction, No. (%)	63 (0.5)	57 (0.5)	32 (0.4)	12 (0.5)	13 (1)	.03
6-mo serum creatinine level, mean (SD), mg/dL^b^	1.41 (0.64)	1.46 (0.68)	1.46 (0.66)	1.50 (0.74)	1.40 (0.65)	<.001
6-mo eGFR, mean (SD), mL/min/1.73 m^2^^b^	61 (22)	58 (22)	58 (22)	57 (22)	62 (22)	<.001

Abbreviations: AKI, acute kidney injury; eGFR, estimated glomerular filtration rate.

SI conversion factor: To convert serum creatinine level to micromoles per liter, multiply by 88.4.

^a^ *P* value compares deceased donors without AKI against stages of AKI.

^b^ Six-month renal function is missing in 2693 recipients.

Death-censored graft failure was comparable by deceased donor AKI status, with an HR of 1.01 (95% CI, 0.95-1.08). When further examined by stages of AKI (Table 3), there was no substantial risk for dcGF, with HRs of 1.03 (95% CI, 0.96-1.11), 1.01 (95% CI, 0.91-1.13), and 0.94 (95% CI, 0.81-1.11) for stages 1, 2, and 3, respectively, compared with kidneys from deceased donors without AKI. Kaplan-Meier curves for dcGF showed no statistically significant difference in survival by AKI stage (log-rank *P* = .69) (eFigure 2A in the Supplement). The results were consistent after examining by AKI stage and adjusting for recipient and transplant characteristics.

**Table 3.** Graft Failure Risk by Deceased Donor AKI

Variable	Deceased Donor AKI	No. of Events/No. of Recipients	Event Rate, Mean (95% CI) per 1000 Person-Years	Hazard Ratio (95% CI)	
				Unadjusted	Adjusted^a^
Death-censored graft failure	No AKI	1809/12 810	30.9 (29.5-32.4)	1 [Reference]	1 [Reference]
	Stage 1	1269/8627	32.2 (30.5-34.0)	1.03 (0.96-1.11)	1.03 (0.96-1.11)
	Stage 2	375/2613	31.5 (28.4-34.9)	1.01 (0.91-1.13)	1.00 (0.89-1.12)
	Stage 3	169/1273	27.9 (23.9-32.7)	0.94 (0.81-1.11)	0.90 (0.77-1.06)
All-cause graft failure	No AKI	3487/12 810	60.5 (58.5-62.5)	1 [Reference]	1 [Reference]
	Stage 1	2410/8627	61.8 (59.4-64.4)	1.01 (0.96-1.07)	0.99 (0.94-1.05)
	Stage 2	721/2613	61.5 (57.1-66.2)	1.01 (0.93-1.10)	0.96 (0.89-1.05)
	Stage 3	313/1273	53.5 (47.8-59.9)	0.91 (0.81-1.02)	0.85 (0.75-0.95)

Abbreviation: AKI, acute kidney injury.

^a^ Adjusted for cold ischemia time and the following recipient variables: age, sex, black race, diabetes as the cause of recipient end-stage renal disease, preemptive transplant, previous kidney transplant, HLA mismatch level, panel reactive antibody (percentage), and body mass index.

All-cause GF also did not differ by deceased donor AKI status, with an HR of 0.97 (95% CI, 0.93-1.02). Further analyzing by AKI stage (Table 3), there was no substantial risk for all-cause GF, with HRs of 1.01 (95% CI, 0.96-1.07), 1.01 (95% CI, 0.93-1.10), and 0.91 (95% CI, 0.81-1.02) for stages 1, 2, and 3, respectively, compared with kidneys from deceased donors without AKI. There were also no statistically significant differences in Kaplan-Meier curves by AKI stage (log-rank *P* = .32) (eFigure 2B in the Supplement). Stage 1 and stage 2 AKI were not associated with all-cause GF after adjustment for recipient and transplant characteristics. After adjustment for recipient and transplant characteristics, stage 3 AKI was associated with reduced risk for all-cause GF (HR, 0.85; 95% CI, 0.75-0.95) compared with no AKI.

### Inverse Probability Treatment Weighting Analysis

The results from inverse probability treatment weighting among the entire cohort are consistent with the results from the propensity score–matched analysis (eTable 5 in the Supplement). The adjusted HRs for dcGF were 1.05 (95% CI, 0.98-1.12), 1.04 (95% CI, 0.93-1.17), and 0.92 (95% CI, 0.77-1.08) for AKI stages 1, 2, and 3, respectively, compared with kidneys from deceased donors without AKI. The adjusted HRs for all-cause GF were 1.01 (95% CI, 0.96-1.06), 1.00 (95% CI, 0.92-1.09), and 0.87 (95% CI, 0.77-0.99) for AKI stages 1, 2, and 3, respectively, compared with kidneys from deceased donors without AKI.

### AKI Kidney Allocation in the United States

From 10 275 deceased donors with AKI, 17 468 of 20 550 kidneys (85%) were recovered during the study period, and 12 711 AKI kidneys were transplanted. As a result, 3030 kidneys from deceased donors with AKI were never procured, and 4757 of 17 468 (27%) AKI kidneys were discarded after recovery. Kidneys with AKI accounted for almost half (4757 of 10 075) of all discarded kidneys during this period. The median proportions of recovery and subsequent discard were 87.7% (IQR, 81.9%-91.5%) and 26.1% (IQR, 21.5%-29.8%), respectively. A scatterplot of all recovery and discard proportions of AKI kidneys by OPOs is shown in eFigure 3 in the Supplement. Comparing discarded and transplanted kidneys by deceased donor AKI status (eTable 6 in the Supplement), donor age and donor cause of death by cerebrovascular accident or stroke differed notably by deceased donor AKI status.

**Figure 2.**
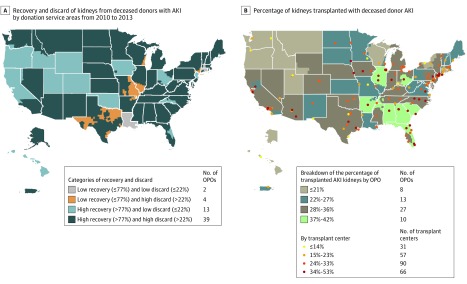
Allocation of Kidneys With Acute Kidney Injury (AKI) in the United States A, Each organ procurement organization (OPO) is responsible for coordinating the donation processes in their corresponding donation service area. The recovery and discard proportions are calculated at the OPO level and mapped at their corresponding donation service area level. B, The percentage of AKI kidneys transplanted of the total number of kidneys transplanted is calculated at both the OPO level and the transplant center level and mapped.

Among the 58 OPOs from 2010 to 2013, the lowest tertile of recovery was 77%, and the lowest tertile of discard was 22%. Figure 2A shows the distribution of allocation practices for AKI kidneys by OPO based on the 4 categories of recovery and discard. Most OPOs (39 of 58) had high recovery and high discard of AKI kidneys. Thirteen OPOs had high recovery and low discard of AKI kidneys. Four OPOs had low recovery and high discard of AKI kidneys, and 2 OPOs had low recovery and low discard of AKI kidneys. Although transplants of AKI kidneys was also highly variable across OPOs and transplant centers, the distribution appears to be largely geographically consistent with the availability of AKI kidneys (Figure 2B). A higher proportion of stage 3 AKI kidneys (19%) were more likely to be offered nationally than kidneys from deceased donors without AKI or with lesser stages of AKI (eTable 7 in the Supplement).

## Discussion

In this registry-based, propensity score–matched study, deceased donor AKI was associated with increased risk for DGF but not with dcGF or all-cause GF. These findings were consistent when further examined by stages of AKI. Despite the evidence in support of transplanting selected AKI kidneys, there is variation in the recovery and transplant of AKI kidneys. The transplant community may need to consider innovative strategies and research regarding the use of kidneys from deceased donors with AKI in settings of additional comorbidities that are typically associated with kidney discard.

Our findings for DGF are consistent with prior studies^17,46^ that indicate deceased donor AKI is associated with increased risk for DGF. Differences in risk were observed for PNF, but rates remained 1% or less across all stages of AKI. No differences were observed for dcGF and all-cause GF. Although DGF requires interventions (eg, additional dialysis sessions, modified initial immunosuppression regimens, and closer recipient monitoring) during initial follow-up,^47^ the lack of sustained risk suggests that recovery after DGF is comparable between AKI and non-AKI kidneys, without substantial long-term sequelae.

In this study, associations of deceased donor AKI with graft outcomes were consistent across stages of AKI, which is largely consistent with prior studies.^17,18,19,20,21,22,23,24,25,26^ A detailed review of more than 585 AKI kidneys transplanted from 2010 to 2013 across 13 transplant centers showed comparable outcomes, with 1845 kidneys that did not have AKI, even at the highest stages of deceased donor AKI.^17^ These findings are congruent with the pooled findings in a systematic review and meta-analysis by Zheng et al,^46^ who found that recipient graft survival of 22 487 kidneys across 14 cohorts was indistinguishable by deceased donor AKI status. The only exception within the meta-analysis was a registry study^48^ from the United Kingdom, which found modestly inferior recipient outcomes after transplant of stage 3 AKI kidneys. However, the kidney discard rate in the United Kingdom is 150% lower than that in the United States (10% vs 25%),^48^ suggesting that the elevated risk seen with stage 3 AKI kidneys, if present, is limited to a small subgroup with high severity of disease.

In light of the overall evidence supporting the use of AKI kidneys across studies,^17,18,19,20,21,22,23,24,25,26^ the concerns raised by the registry study^48^ in the United Kingdom regarding stage 3 AKI kidneys should not deter recovery and transplants of AKI kidneys. Instead, the findings from the United Kingdom should prompt additional safeguards and development of interventions to ensure allograft success. Examples include caution when transplanting AKI kidneys into recipients sensitive to volume overload and risk of heart failure, careful review of procurement biopsy specimens to avoid AKI kidneys with extensive cortical necrosis, and further research into ischemic preconditioning,^49^ better repair and recovery biomarkers,^13^ and development of donor-level interventions before nephrectomy and transplant.^47^ Our results indicate a slightly protective association of stage 3 AKI for all-cause GF, which is most likely explained by increased precautions and closer follow-up. Furthermore, stage 3 accounts for less than 20% of deceased donor AKI in the United States. The transplant community should embrace the use of the 80% of AKI kidneys with less severe injury (stage 1 or stage 2), while advancing transplants of stage 3 AKI kidneys with appropriate safeguards and considerations.

Another important finding of our study is that AKI kidney allocation practices vary by OPO. Although there is no consensus regarding the allocation of AKI kidneys, these kidneys can be carefully incorporated into the transplant system through pilot studies, such as the Collaborative Improvement and Innovation Network (COIIN) project. In 2017, a total of 19 transplant centers participated in the first phase of COINN,^50^ followed by a second cohort of 36 transplant centers,^51^ to evaluate and increase the use of high-KDPI kidneys. Stewart et al^52^ recently used SimUNet, a DonorNet offer simulator, to investigate whether biopsy findings substantially and variably alter kidney offer acceptance decisions for deceased donors with and without AKI. Additional SimUNet approaches could be considered to help further understand the consequences of deceased donor AKI. For example, acceptance rates for deceased donor AKI kidneys that provide KDPIs based on admission Scr levels could be compared with current KDPIs that use the terminal Scr level alone.

### Strengths and Limitations

Our study has several strengths. First, this is a large, registry-based study encompassing all kidney transplants in the United States over a 3-year period. Prior studies have been limited by small sample sizes, particularly for the number of deceased donors with stage 3 AKI. In the United Kingdom registry,^48^ only 172 deceased donors had stage 3 AKI from 2003 to 2013. In our propensity score–matched analysis, 692 deceased donors from 2010 to 2013 had stage 3 AKI. Second, we confirmed our findings with the entire cohort through inverse probability treatment weighting, which provides reassurance for the generalizability of our findings. Third, DonorNet provides granular information on admission Scr level, which allowed rigorous clinical classification of AKI and its severity that is more consistent with current international guidelines.^35^

There are several limitations of our study to consider. First, our results do not provide the true strength of a randomized clinical trial. However, propensity score methods are a popular tool to align baseline characteristics between groups similar to randomization in clinical trials.^37^ Second, our maps are based on DonorNet data from 2010 to 2013. In December 2014, the KAS was introduced,^15^ and deceased donor kidney transplantation has increased since its implementation. However, the KAS did not substantially alter kidney discard rates, which remained approximately 20%.^14^ Third, posttransplant kidney function data beyond 6 months were not available. Fourth, our calculations for recovery and discard only capture AKI-associated recovery and discard. The OPO-level decisions are multifactorial and depend on the availability of transplant centers willing to accept the kidneys in question. In fact, a large number of deceased donor kidney offers are declined on behalf of transplant candidates.^53^ Our analysis does not account for the other factors and regulatory bodies involved in transplant decisions.

## Conclusions

This study found that the AKI kidneys transplanted in the United States from 2010 to 2013 had comparable rates of recipient graft survival, even among the highest stages of injury. There was OPO-level variation in the allocation practices of AKI kidneys. From our study’s findings, we believe that the transplant community should continue to use deceased donor AKI kidneys and consider research to investigate whether currently discarded AKI kidneys from deceased donors without substantial comorbidities can be used more effectively.

## Notes

Supplement. **eTable 1.** Validation of Serum Creatinine Levels in DonorNet Data With Chart Abstracted Data From the Deceased Donor Study **eTable 2.** Evaluate Balance of Donor Characteristics in Propensity Score Analyses **eTable 3.** Matching Criteria Characteristics of Unmatched Donors With and Without AKI **eTable 4.** Donor and Recipient Characteristics by Donor AKI Stage in Propensity Score–Matched Analysis **eTable 5.** Graft Failure Risk by Donor AKI From the Propensity Score Inverse Probability Weighting Analysis **eTable 6.** Donor Characteristics by Discard and AKI Status **eTable 7.** Breakdown of Geographical Distribution of Transplanted Kidneys **eFigure 1.** Comparing Distribution of Continuous Covariates in Propensity Score–Matched Sample **eFigure 2.** Kaplan-Meier Curves of Death-Censored Graft Survival and All-Cause Graft Survival by Donor AKI Stage **eFigure 3.** Scatterplot of Recovery and Discard Proportions of AKI Kidneys by Organ Procurement Organization, Weighted by the Number of AKI Kidneys Available for Recovery
