# Supplementary material for: Association of Deceased Donor Acute Kidney Injury With Recipient Graft Survival
Source: JAMA Netw Open. 2020 Jan 8;3(1):e1918634. doi: 10.1001/jamanetworkopen.2019.18634 (PMC6991314; doi:10.1001/jamanetworkopen.2019.18634)
Supplement: Supplement. — eTable 1. Validation of Serum Creatinine Levels in DonorNet Data With Chart Abstracted Data From the Deceased Donor Study eTable 2. Evaluate Balance of Donor Characteristics in Propensity Score Analyses eTable 3. Matching Criteria Characteristics of Unmatched Donors With and Without AKI eTable 4. Donor and Recipient Characteristics by Donor AKI Stage in Propensity Score–Matched Analysis eTable 5. Graft Failure Risk by Donor AKI From the Propensity Score Inverse Probability Weighting Analysis eTable 6. Donor Characteristics by Discard and AKI Status eTable 7. Breakdown of Geographical Distribution of Transplanted Kidneys eFigure 1. Comparing Distribution of Continuous Covariates in Propensity Score–Matched Sample eFigure 2. Kaplan-Meier Curves of Death-Censored Graft Survival and All-Cause Graft Survival by Donor AKI Stage eFigure 3. Scatterplot of Recovery and Discard Proportions of AKI Kidneys by Organ Procurement Organization, Weighted by the Number of AKI Kidneys Available for Recovery [file jamanetwopen-3-e1918634-s001.pdf]

Supplementary Online Content

Liu C, Hall IE, Mansour S, Thiessen Philbrook HR, Jia Y, Parikh CR. Association of deceased donor acute kidney injury with recipient graft survival. *JAMA Netw Open*. 2020;3(1):e1918634 doi:10.1001/jamanetworkopen.2019.18634

eTable 1. Validation of Serum Creatinine Levels in DonorNet Data With Chart Abstracted Data From the Deceased Donor Study

eTable 2. Evaluate Balance of Donor Characteristics in Propensity Score Analyses

eTable 3. Matching Criteria Characteristics of Unmatched Donors With and Without AKI

eTable 4. Donor and Recipient Characteristics by Donor AKI Stage in Propensity Score–Matched Analysis

eTable 5. Graft Failure Risk by Donor AKI From the Propensity Score Inverse Probability Weighting Analysis

eTable 6. Donor Characteristics by Discard and AKI Status

eTable 7. Breakdown of Geographical Distribution of Transplanted Kidneys

eFigure 1. Comparing Distribution of Continuous Covariates in Propensity Score–Matched Sample

eFigure 2. Kaplan-Meier Curves of Death-Censored Graft Survival and All-Cause Graft Survival by Donor AKI Stage

eFigure 3. Scatterplot of Recovery and Discard Proportions of AKI Kidneys by Organ Procurement Organization, Weighted by the Number of AKI Kidneys Available for Recovery

This supplementary material has been provided by the authors to give readers additional information about their work.

eTable 1. Validation of Serum Creatinine Levels in DonorNet data With Chart Abstracted Data From the Deceased Donor Study

The Deceased Donor Study is a multicenter, observational study of deceased kidney donors and their kidney recipients. Five organ procurement organizations enrolled 1679 deceased donors from 2010-2013. Clinical details were abstracted from medical health records. In a subset of 424 donors, we abstracted daily lab values from admission to procurement. If multiple lab values were available on the same day, we abstracted the highest value for BUN and Creatinine, and selected the ABG variables with the lowest pH. We compared the abstracted serial lab data with lab data in DonorNet. For each daily value abstracted, we compared with the daily value report in DonorNet. Of the 1,980 abstract serum creatinine values, 97% (1918) values were identical between data sources, 1% (23) reported a different serum creatinine on the given date, and 2% (39) did not have a value in DonorNet reported on the given date. Other lab tests (such as BUN, PaCO2, PaO2, HCO3) were identically matched in 97% of tests and SaO2 was matched in 20% of tests.

**Lab Test**	**# tests**	**Exact Matched**	**Date Matched**	**No match**
		**value and date identical**	**different value on date**	**value not available on date**
Creatinine (mg/dL)	1980	1918 (97%)	23 (1%)	39 (2%)
BUN (mg/dL)	1978	1918 (97%)	21 (1%)	60 (3%)
PaCO2 (mmGh)	1645	1595 (97%)	35 (2%)	15 (1%)
PaO2 (mmHg)	1642	1589 (97%)	14 (1%)	39 (2%)
HCO3 (mEq/L)	1638	1582 (97%)	20 (1%)	56 (3%)
SaO2 %	1351	265 (20%)	772 (57%)	314 (23%)

© 2020 Liu C et al. *JAMA Network Open*.

eTable 2. Evaluate Balance of Donor Characteristics in Propensity Score Analyses

	Entire Study Cohort			Propensity Score Matched Cohort			Propensity Score IPTW Cohort		
**Donor Characteristics**	**No AKI (n_d=15,310)**	**AKI (n_d=6,832)**	**SD (%)**	**Donors without AKI (n_d=6,722)**	**Donors with AKI (n_d=6,722)**	**SD (%)**	**Donors without AKI (n_d=15,310)**	**Donors with AKI (n_d=6,832)**	**SD (%)**
Age, years	39 (14)	40 (14)	-9.4	40 (15)	40 (14)	0.5	40 (15)	40 (14)	2.2
Male sex	9061 (59%)	4342 (64%)	-9.0	4265 (63%)	4264 (63%)	0	9402 (60%)	3983 (60%)	0.4
Black race	1846 (12%)	1313 (19%)	-19.8	1324 (20%)	1227 (18%)	3.7	2205 (14%)	928 (14%)	0.4
BMI, kg/m^2	27.53 (6.27)	28.52 (6.97)	-14.9	28.52 (6.97)	28.34 (6.72)	2.7	27.83 (6.55)	27.80 (6.42)	0.5
Hypertension	4038 (26%)	2276 (33%)	-15.2	2204 (33%)	2191 (33%)	0.4	4404 (28%)	1820 (28%)	1.8
Diabetes	1068 (7%)	615 (9%)	-7.5	572 (9%)	579 (9%)	-0.4	1172 (8%)	485 (7%)	0.8
DCD	2357 (15%)	488 (7%)	26.3	569 (8%)	487 (7%)	4.5	1999 (13%)	864 (13%)	-0.7
Hepatitis C virus	478 (3%)	155 (2%)	5.3	155 (2%)	155 (2%)	0	443 (3%)	181 (3%)	0.7
Admission SCr, mg/dL	1.03 (0.42)	0.98 (0.4)	13.0	0.98 (0.35)	0.98 (0.4)	1.1	1.01 (0.40)	1.03 (0.49)	-4.5

All values are mean (standard deviation) or n (%)
Abbreviations: AKI, acute kidney injury; BMI, body mass index; DCD, donation after cardiac determination of death; IPTW, inverse probability treatment weighting; SCr, serum creatinine, SD; standardized difference.

© 2020 Liu C et al. *JAMA Network Open*.

eTable 3. Matching Criteria Characteristics of Unmatched Donors With and Without AKI

	**No AKI (N=8588)**	**AKI (N=110)**
Age, years	38 (25, 50)	47(38.5,54.5)
Male sex	4789 (56%)	76 (73%)
Black race	518 (6%)	86 (83%)
Body mass index, kg/m^2	25.89 (22.79, 29.83)	36.85 (29.96, 49.21)
Hypertension	1830 (21%)	84 (81%)
Diabetes	495 (6%)	35 (34%)
DCD	1781 (21%)	0
Hepatitis C virus	322 (4%)	0
Admission SCr, mg/dL	1 (0.8, 1.26)	0.84 (0.65, 1)

All values are median (IQR) or n (%)

Abbreviations: AKI, acute kidney injury; DCD, donation after cardiac determination of death; SCr, serum creatinine.

© 2020 Liu C et al. *JAMA Network Open*.

eTable 4. Donor and Recipient Characteristics by Donor AKI Stage in Propensity Score–Matched Analysis

	Donors without AKI (n_d=6,722)	Donors with Stage 1 AKI (n_d=4,621)	Donors with Stage 2 AKI (n_d=1,409)	Donors with Stage 3 AKI (n_d=692)
**Donor Characteristics**				
Age, years	42 (27, 52)	43 (28, 52)	42 (29, 52)	34 (25, 45)
Male sex	4265 (63%)	3012 (65%)	821 (58%)	431 (62%)
Black race	1324 (20%)	859 (19%)	268 (19%)	100 (14%)
Body mass index, kg/m^2	27.27 (23.65, 31.85)	27.1 (23.63, 31.57)	27.17 (23.53, 31.46)	27.84 (24.26, 32.63)
Hypertension	2204 (33%)	1518 (33%)	504 (36%)	169 (24%)
Diabetes	572 (9%)	408 (9%)	128 (9%)	43 (6%)
Donation after cardiac death	569 (8%)	337 (7%)	117 (8%)	33 (5%)
Expanded Criteria donor[a]	1216 (18%)	939 (20%)	307 (22%)	70 (10%)
Hepatitis C Serostatus	155 (2%)	130 (3%)	20 (1%)	5 (1%)
Admission serum creatinine, mg/dL	0.9 (0.74, 1.16)	0.97 (0.76, 1.2)	0.8 (0.6, 1.05)	0.91 (0.64, 1.39)
CDC high risk status	716 (11%)	515 (11%)	161 (11%)	110 (16%)
Duration of AKI, days	0 (0, 0)	2 (1, 2)	2 (2, 3)	3 (2, 4)
Terminal serum creatinine, mg/dL	0.8 (0.7, 1)	1.3 (1.08, 1.7)	1.8 (1.3, 2.39)	3.5 (2.2, 4.73)
Number of kidneys transplanted				
1	634 (9%)	615 (13%)	204 (14%)	111 (16%)
2	6088 (91%)	4006 (87%)	1205 (86%)	581 (84%)
Kidney Donor Risk Index	1.14 (0.9, 1.46)	1.25 (1, 1.58)	1.3 (1.06, 1.61)	1.17 (0.99, 1.39)
Kidney Donor Profile Index	41 (19, 66)	51 (29, 74)	55 (35, 75)	44 (28, 62)
0-20%	1744 (26%)	646 (14%)	144 (10%)	80 (12%)
20-50%	2201 (33%)	1579 (34%)	461 (33%)	315 (46%)
50-85%	2194 (33%)	1781 (39%)	591 (42%)	251 (36%)
85-100%	583 (9%)	615 (13%)	213 (15%)	46 (7%)
Cause of death				
Anoxia	1478 (22%)	1066 (23%)	359 (25%)	283 (41%)
Cerebrovascular/stroke	2353 (35%)	1788 (39%)	562 (40%)	194 (28%)
Head trauma	2692 (40%)	1674 (36%)	443 (31%)	198 (29%)
Other	199 (3%)	93 (2%)	45 (3%)	17 (2%)
Number of days from donor admission to organ recovery	3 (2,5)	3 (2,4)	3 (2,5)	4 (3.6)
**Recipient Characteristics**	**n_r=12,810**	**n_r=8,627**	**n_r=2,613**	**n_r=1,273**
Age				
<18 years	525 (4%)	235 (3%)	39 (1%)	17 (1%)

© 2020 Liu C et al. *JAMA Network Open*.

18-29 years	627 (5%)	384 (4%)	114 (4%)	54 (4%)
30-39 years	1375 (11%)	908 (11%)	258 (10%)	143 (11%)
40-49 years	2520 (20%)	1651 (19%)	516 (20%)	249 (20%)
50-59 years	3488 (27%)	2330 (27%)	718 (27%)	338 (27%)
≥60 years	4275 (33%)	3119 (36%)	968 (37%)	472 (37%)
Male sex	7821 (61%)	5264 (61%)	1571 (60%)	829 (65%)
Black race	4071 (32%)	2830 (33%)	877 (34%)	402 (32%)
Wait time				
<6 months	2521 (20%)	1650 (19%)	410 (16%)	211 (17%)
6 months <2 years	3778 (29%)	2477 (29%)	729 (28%)	343 (27%)
≥2 years	6511 (51%)	4500 (52%)	1474 (56%)	719 (56%)
Recipient ESKD cause				
Diabetes	4085 (32%)	2774 (32%)	797 (31%)	367 (29%)
Hypertension	2884 (23%)	2004 (23%)	634 (24%)	339 (27%)
Glomerulonephritis	2085 (16%)	1350 (16%)	422 (16%)	222 (17%)
Graft failure	945 (7%)	557 (6%)	184 (7%)	76 (6%)
Other or unknown	2811 (22%)	1942 (23%)	576 (22%)	269 (21%)
Preemptive transplant	1595 (12%)	994 (12%)	327 (13%)	110 (9%)
Previous kidney transplant	1556 (12%)	959 (11%)	281 (11%)	122 (10%)
Pretransplant blood transfusion	2361 (18%)	1625 (19%)	501 (19%)	203 (16%)
HLA mismatch level				
0	900 (7%)	626 (7%)	161 (6%)	51 (4%)
1	128 (1%)	81 (1%)	26 (1%)	7 (1%)
2	503 (4%)	338 (4%)	85 (3%)	60 (5%)
3	1592 (13%)	1089 (13%)	328 (13%)	177 (14%)
4	3362 (26%)	2235 (26%)	699 (27%)	347 (27%)
5	4133 (32%)	2809 (33%)	886 (34%)	399 (32%)
6	2103 (17%)	1387 (16%)	409 (16%)	224 (18%)
PRA				
0-9%	8640 (67%)	5876 (68%)	1822 (70%)	953 (75%)
10-39%	1161 (9%)	822 (10%)	226 (9%)	94 (7%)
40-84%	1573 (12%)	1007 (12%)	281 (11%)	151 (12%)
≥85%	1436 (11%)	922 (11%)	284 (11%)	75 (6%)
ESKD duration, months	42.15 (22.08, 66.27)	42.78 (22.37, 67.71)	45.65 (25.69, 70.7)	45.18 (24.77, 69.69)
Kidney biopsied	5346 (42%)	4541 (53%)	1719 (66%)	1039 (82%)
Kidney pumped	4044 (32%)	3069 (36%)	1090 (42%)	549 (43%)
Cold ischemia time, hours	14.33 (9.88, 20.02)	15.42 (10.5, 21.5)	16.89 (11.52, 23.28)	18.17 (13, 26.2)

All values are median (IQR) or n (%)

© 2020 Liu C et al. *JAMA Network Open*.

Abbreviations: AKI, acute kidney injury; CDC, Centers for Disease Control and Prevention; CI, confidence interval; ESKD, end stage kidney disease.
[a] Expanded Criteria Donor defined as donor age ≥60 years or 50-59 years with two or more of the following risk factors: terminal serum creatinine > 1.5mg/dL, history of hypertension, stroke as the cause of death.

© 2020 Liu C et al. *JAMA Network Open*.

eTable 5. Graft Failure Risk by Donor AKI From the Propensity Score Inverse Probability Weighting Analysis

Outcome	Donor AKI	Events / Total n_r	Unadjusted Hazard Ratio (95% CI)	Adjusted[a] Hazard Ratio (95% CI)
Death Censored Graft Failure	No AKI	4021/29583	1 (ref)	1 (ref)
	Stage 1	1210/8447	1.05 (0.98, 1.12)	1.05 (0.98, 1.12)
	Stage 2	357/2490	1.06 (0.95, 1.19)	1.04 (0.93, 1.17)
	Stage 3	174/1353	0.96 (0.81, 1.13)	0.92 (0.77, 1.08)
All-Cause Graft Failure	No AKI	7726/29583	1 (ref)	1 (ref)
	Stage 1	2307/8447	1.04 (0.99, 1.09)	1.01 (0.96, 1.06)
	Stage 2	678/2490	1.05 (0.97, 1.14)	1.00 (0.92, 1.09)
	Stage 3	324/1353	0.93 (0.82, 1.06)	0.87 (0.77, 0.99)

Abbreviations: AKI, acute kidney injury; CI, confidence interval.

[a]Adjusted for cold ischemia time, and the following recipient variables: age (years), black race, sex, previous kidney transplant, diabetes as the cause of end-stage kidney disease, number of human leukocyte antigen mismatches, panel reactive antibody (%), body mass index (kg/m^2), and pre-emptive transplant

© 2020 Liu C et al. *JAMA Network Open*.

eTable 6. Donor Characteristics by Discard and AKI Status

	AKI			No AKI		
**Donor Characteristics**	**Discarded (N=3443)**	**Transplanted (N=6832)**	**SD(%)[a]**	**Discarded (N=2657)**	**Transplanted (N=15310)**	**SD(%)[a]**
**Age (years)**	52 (14)	40 (14)	85.4	56 (14)	39 (14)	119.4
**Male, n (%)**	1981 (58%)	4342 (64%)	-12.3	1295 (49%)	9061 (59%)	21.1
**Black, n (%)**	926 (27%)	1313 (19%)	18.3	406 (15%)	1846 (12%)	9.4
**Body mass index (kg/m^2)**	29.74 (7.43)	28.52 (6.97)	17.0	28.7 (7.14)	27.53 (6.27)	17.3
**Hypertension, n (%)**	2298 (67%)	2276 (33%)	70.9	1823 (69%)	4038 (26%)	93.3
**Diabetes, n (%)**	1024 (30%)	615 (9%)	54.4	790 (30%)	1068 (7%)	61.5
**Donation after cardiac death, n (%)**	130 (4%)	488 (7%)	-14.9	279 (11%)	2357 (15%)	-14.6
**Baseline serum creatinine (mg/dL)**	1.56 (1.24)	0.98 (0.4)	63.5	1.52 (1.5)	1.03 (0.42)	44.2
**Baseline serum creatinine ≥ 1.5 mg/dL**	1181 (34%)	645 (9%)	63.1	700 (26%)	1743 (11%)	39.0
**Kidney Donor Profile Index (KDPI)**	78.83 (21.55)	51.83 (25.95)	113.2	78.17 (21.26)	40.31 (26.86)	156.3
**Categories of KDPI**						
0-20%	56 (2%)	872 (13%)	-44.1	63 (2%)	4393 (29%)	-78.0
20-50%	368 (11%)	2369 (35%)	-59.8	240 (9%)	5260 (34%)	-64.6
50-85%	1186 (34%)	2674 (39%)	-9.7	1000 (38%)	4658 (30%)	15.3
85-100%	1831 (53%)	915 (13%)	93.2	1352 (51%)	988 (6%)	112.9
**Kidney Donor Risk Index**	1.94 (0.62)	1.34 (0.41)	114.0	1.9 (0.58)	1.18 (0.37)	146.8
**Terminal serum creatinine (mg/dL)**	3.11 (2.14)	1.75 (1.2)	78.2	1.31 (1.29)	0.89 (0.61)	41.6
**Donor cause of death**						
Anoxia	1026 (30%)	1731 (25%)	10.0	683 (26%)	4210 (27%)	-4.1
Cerebrovascular/stroke	1854 (54%)	2614 (38%)	31.7	1524 (57%)	4636 (30%)	56.7
Head trauma	497 (14%)	2326 (34%)	-47.0	375 (14%)	6013 (39%)	-59.3
Other	66 (2%)	161 (2%)	-3.0	75 (3%)	451 (3%)	-0.7
**Days from donor admission to organ recovery**	4.36 (11.45)	8.57 (148.54)	-4.0	9.24 (234.25)	5.96 (46.38)	1.9
**Number of kidneys pumped**						
0	2837 (82%)	3955 (58%)	55.6	2035 (77%)	9513 (62%)	31.7
1	66 (2%)	642 (9%)	-32.8	73 (3%)	1490 (10%)	-29.2
2	540 (16%)	2235 (33%)	-40.6	549 (21%)	4307 (28%)	-17.5

© 2020 Liu C et al. *JAMA Network Open*.

**Number of kidneys biopsied**						
0	1684 (49%)	2623 (38%)	21.3	891 (34%)	8955 (58%)	-51.7
1	72 (2%)	239 (3%)	-8.5	66 (2%)	439 (3%)	-2.4
2	1687 (49%)	3970 (58%)	-18.3	1700 (64%)	5916 (39%)	52.4

All values are mean (standard deviation) or n (%)
Abbreviations: AKI, acute kidney injury; KDPI, kidney donor profile index; SD, standardized difference.
[a] Grey highlighted cells indicate SDs that were more 25% different between the No AKI and AKI group. These highlight donor characteristics that are notably differences between No AKI and AKI kidneys when comparing discarded vs. transplanted kidneys

© 2020 Liu C et al. *JAMA Network Open*.

eTable 7. Breakdown of Geographical Distribution of Transplanted Kidneys

Share Type	No AKI (n=32,308)	AKI (n=13,608)	Stage 1 AKI (n=9,240)	Stage 2 AKI (n=2,936)	Stage 3 AKI (n=1,432)
Local	80.2%	76.3%	78.7%	73.6%	66.1%
Regional	8.6%	10.4%	9.3%	12.0%	14.7%
National	11.2%	13.3%	12.0%	14.4%	19.2%

Abbreviations: AKI, acute kidney injury

© 2020 Liu C et al. *JAMA Network Open*.

eFigure 1. Comparing Distribution of Continuous Covariates in Propensity–Score Matched Sample

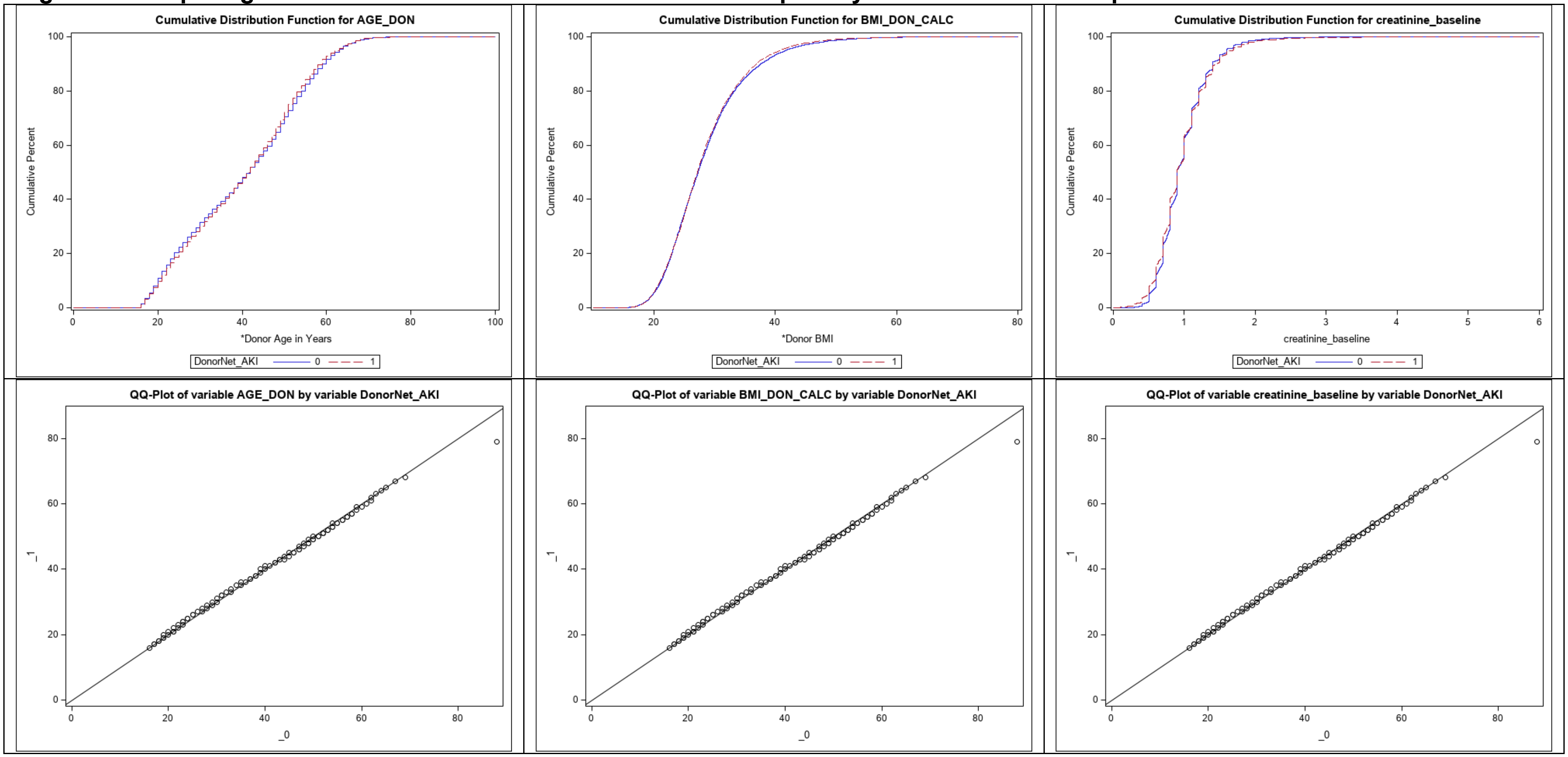

The figures in the top row show cumulative distribution functions for donor age, body mass index, and baseline serum creatinine by donor AKI status. The figures in the second row shows quantile-quantile plots of donor age, body mass index, and baseline serum creatinine between donors with AKI and donors without AKI.

© 2020 Liu C et al. *JAMA Network Open*.

Figure 2. Kaplan-Meier Curves of Death-Censored Graft Survival and All-Cause Graft Survival by Donor AKI Stage

A. Kaplan Meier of Death-Censored Graft Survival by Donor AKI Stage

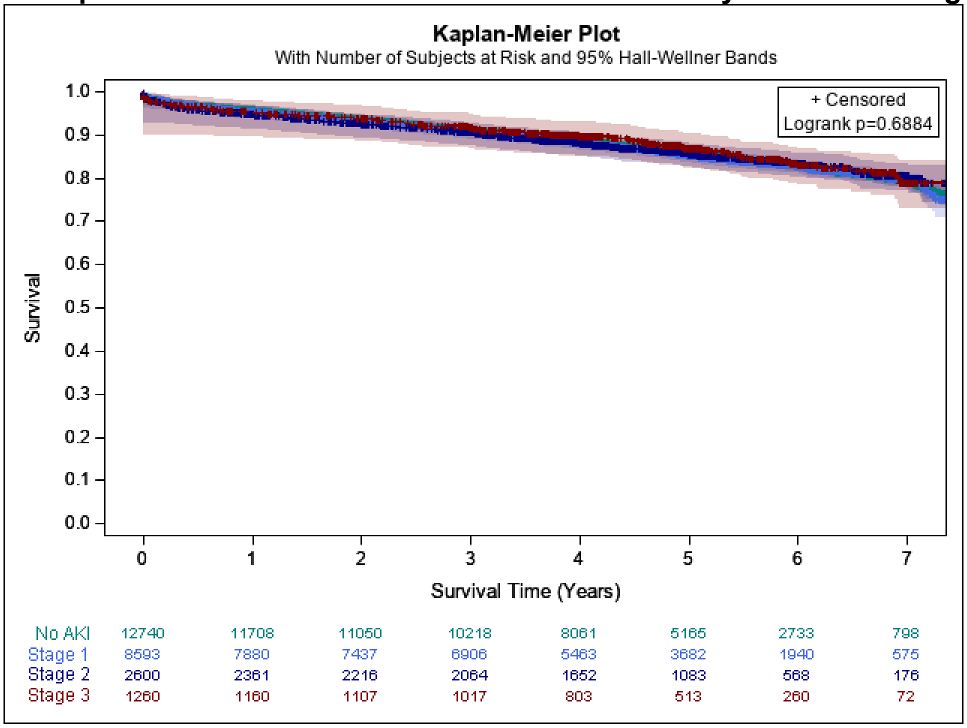

B. Kaplan Meier of All-Cause Graft Survival by Donor AKI Stage

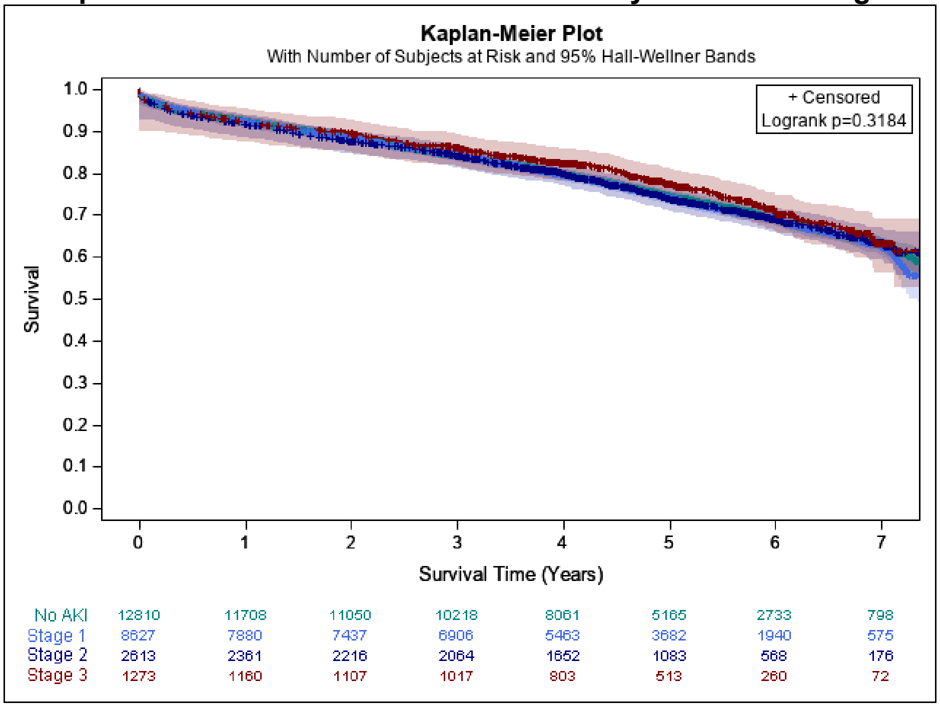

© 2020 Liu C et al. *JAMA Network Open*.

eFigure 3. Scatter Plot of Recovery and Discard Proportions of AKI Kidneys by Organ Procurement Organization, Weighted by the Number of AKI Kidneys Available for Recovery

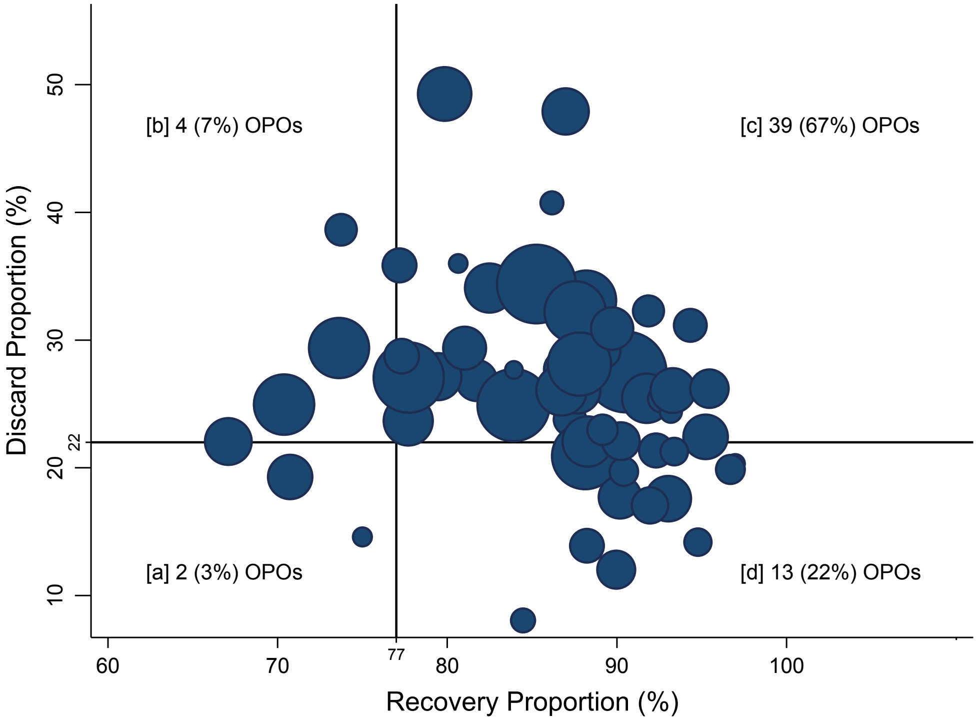

Circles are proportional to the weight of available kidneys from donors with AKI that are available for recovery from 2010-2013. The X and Y line designate lower tertile cutoffs for recovery proportion and discard proportion, respectively. The quadrants represent categories created using the lowest tertile cutoffs and are as follows: a (Low recovery, Low Discard), b (Low Recovery, High Discard), c (High Recovery, High Discard), and d (High Recovery, Low Discard). The n (%) of OPOs that fall into each quadrant are shown in the figure.

© 2020 Liu C et al. *JAMA Network Open*.
